# Association of pasta consumption with body mass index and waist-to-hip ratio: results from Moli-sani and INHES studies

**DOI:** 10.1038/nutd.2016.20

**Published:** 2016-07-04

**Authors:** G Pounis, A Di Castelnuovo, S Costanzo, M Persichillo, M Bonaccio, A Bonanni, C Cerletti, M B Donati, G de Gaetano, L Iacoviello

**Affiliations:** 1 Department of Epidemiology and Prevention, IRCCS Istituto Neurologico Mediterraneo Neuromed, Pozzilli, Italy

## Abstract

**Background/Objectives:**

Pasta as a traditional component of Mediterranean diet (MeD) in Italy has not been studied in detail in the management of body weight. This study aimed at evaluating the association of pasta intake with body mass index (BMI) and waist-to-hip ratio, in two large epidemiological datasets.

**Subjects/Methods:**

A total of 14 402 participants aged ⩾35 years randomly recruited from the general population of the Molise region (Moli-sani cohort) and 8964 participants aged >18 years from all over Italy (Italian Nutrition & HEalth Survey, INHES) were separately analyzed. The European Prospective Investigation into Cancer and Nutrition (EPIC)-food frequency questionnaire and one 24-h dietary recall were used for dietary assessment. Weight, height, waist and hip circumference were measured in Moli-sani or self-reported in INHES. Residuals methodology corrected for either total energy intake or body weight was used for the analysis of pasta intake.

**Results:**

Higher pasta intake was associated with better adhesion to MeD in both genders (*P* for both<0.001). In the Moli-sani study, after multivariable analysis, pasta-energy residuals were negatively associated with BMI in women but not in men (β-coef=−0.007, *P*=0.003 for women and β-coef=−0.001, *P*=0.58 for men). When pasta intake-body weight residuals were used, pasta intake was significantly and negatively associated with BMI in crude and multi-adjusted models (including adhesion to MeD) in both genders and Moli-sani and INHES studies (for all β-coef<0, *P*<0.05). In the Moli-sani study, pasta-body weight residuals were significantly and negatively associated with waist and hip circumference and waist-to-hip ratio (for all β-coef<0, *P*<0.05).

**Conclusions:**

As a traditional component of MeD, pasta consumption was negatively associated with BMI, waist circumference and waist-to-hip ratio and with a lower prevalence of overweight and obesity.

## Introduction

The traditional Mediterranean diet (MeD), a healthy eating behavior model, might be the basis for establishing nutrition guidelines as an outcome of health policies.^1, 2^ Its health benefits in primary and secondary prevention of chronic diseases has long been studied since middle 50s^3, 4, 5^ and confirmed by meta-analysis.^6, 7, 8^

Major components of the MeD are foods with high content of complex carbohydrates and fiber such as legumes, rice and cereals.^1^ The latter represent the main source of carbohydrates in the diet and in Italy, they are consumed mainly in the form of pasta.^9^

In the last decades in Italy, despite the strong effort to promote MeD, a progressive change occurred in eating habits.^10, 11^ Pasta consumption has been decreased,^9, 10, 11, 12, 13, 14^ as a concept of low carbohydrate and high protein diet against obesity emerged. However, the debate of hypo-caloric high protein diets versus low fat and standard carbohydrate diets in the management of body weight and the health implications (that is, kidney function, bone health) is still open.^15, 16, 17, 18, 19, 20^

On the contrary, adherence to the MeD according to epidemiological and clinical evidence has a protective role on overweight and obesity,^21, 22, 23^ in parallel with important health benefits against chronic diseases and related comorbidities.^5, 6^ Despite this fact, the components of MeD have not been studied in deep for their association with body weight and obesity. In particular, epidemiological data about the association of pasta consumption with body mass index (BMI) and prevalence of overweight and obesity are still limited.

This work aimed at evaluating the association of pasta intake with BMI, waist and hip circumference, waist-to-hip ratio, and prevalence of overweight and obesity in the context of MeD adherence. Data from two different large epidemiological studies with different methodology in dietary and anthropometric assessment have been used.

## Subjects and methods

### Study populations

#### Moli-sani participants

The cohort of the Moli-sani Project was randomly recruited in the Molise region (Italy) from city hall registries by a multistage sampling, as previously described.^24, 25^ Between March 2005 and April 2010, 24 325 subjects were enrolled. Participants who had incomplete medical (*n*=235) or dietary questionnaires (*n*=1917) or were not caucasians or not born in Italy (*n*=332) were excluded from the analysis. Furthermore, persons who were under a special diet or a diet for the control of diabetes, hypertension or hyperlipidemia (*n*=6262) were excluded as these conditions may lead to changes in their usual diet. The final study sample included in this analysis consisted of 14 402 subjects (7216 women and 7186 men).

The Moli-sani project was approved by the Catholic University Ethical Committee. All participants provided written informed consent.

#### INHES participants

The Italian Nutrition & Health Survey (INHES) project is a telephone-based survey on nutrition and health specifically designed to collect information on the dietary habits (quality, quantity and patterns), food choice determinants, and food health awareness of the Italian population according to geographical distribution, age, gender and socioeconomic profile. Between November 2010 and November 2013, 9319 women and men aged ⩾5 years from all over Italy were enrolled.

First, subjects (*n*=9106) in the age range 35–79 years, recruited in the 2008–2012 wave of the Cardiovascular Epidemiologic Observatory (participation rate 53%, from 40 to 85% in the different regions)^26, 27^ were invited to participate in the INHES survey. Once they accepted, participants were asked to invite one relative older than 79 or younger than 35 years to join the survey. Finally, 5385 (59.1%) from the original population and 3754 from their relatives were included in the survey.

The sampled subjects were distributed in the four seasons (excluding Christmas, Easter and middle August periods). The survey calendar was organized to capture an adequate proportion of weekdays and weekend days at group level.

The recruitment was performed using computer-assisted telephone interviewing (CATI): 1-day 24-h dietary recall,^9^ the Italian version of the European Food Propensity Questionnaire,^28^ questionnaire of nutrition-related behavior, anamnestic questionnaire on health status, risk factors and anthropometry, and SF12 questionnaire on health perception^29^ were administered.

For the purpose of the present study, the adult (18–96 years) population of 8964 (4782 women and 4182 men) was analyzed. The INHES study was approved by the Ethical Committee of the Catholic University of Rome.

### Dietary assessment

#### Moli-sani population

The European Prospective Investigation into Cancer and Nutrition (EPIC)-food frequency questionnaire, specifically adapted for the Italian population, was used to determine usual nutritional intakes during the previous year.^12^ A computer program, NAF,^30^ was developed by the Epidemiology and Prevention Unit, Fondazione IRCCS, Istituto Nazionale dei Tumori, Milan to convert questionnaire dietary data into frequencies of consumption and average daily amounts of foods (grams per day) and energy intake (kcal per day). NAF was linked to the Italian FTC for the energy assessment.^31^

#### INHES population

Each participant received by mail a short photograph atlas and guidance notes to estimate portion sizes (with instructions to quantify the portions used by children) developed on the basis of EPIC-SOFT picture book^32^ and a hard-copy diary structured by meal, where all the information on food consumption the day before the telephone interview was self-recorded. All foods and drinks consumed (including tap and bottled water), both at and outside home, were recorded. The day after, participants were interviewed by telephone by trained and standardized interviewers, starting from the self-recorded diary, by using a computer-based 24-h dietary recall interview software.

For every eating occasion, subjects were asked to carefully record and recall: time, place of consumption, detailed description of foods (or beverages), quantity consumed and brand (for manufactured foods). Portion sizes were reported by subjects with the help of a picture booklet. Moreover, it was asked whether they were following a particular diet and whether the consumption they had reported differed from their usual consumption.

The data management system INRAN-DIARIO 3.1 developed by INRAN^9, 33^ in previous surveys^9, 34^ was used for data coding, data entry and data processing. This software includes several checkpoints to ensure the accuracy and completeness of the data recorded and allows each interviewer to create new temporary food codes for all the food items and recipes that are not present in the databanks. Four databases were used to transform the data reported by subjects into the weight of single foods, raw ingredients and into the amounts of nutrients consumed. The portions estimated by subjects with the help of the picture booklet are linked to the specific weight of each food item. This database contains a total of 9450 entries (weight of standard portions of specific dishes or units of measurement) for 2460 foods, that is, on average approximately four entries per food. Any missing food consumed during the survey was added to the food composition database.

For both studies, pasta consumption was calculated and expressed as grams per day and g kcal^−1^ of daily energy intake. Adherence to MeD was evaluated by applying a dietary score ranging from 0 to 11, that have been specifically developed for the Italian population.^34^ The increase in that score was associated with higher adherence to MeD.

### Measurements and definition of factors

#### Moli-sani population

Socioeconomic status was defined as a score based on eight variables ranging from 0 to 8; the higher the score, the higher the level of socioeconomic status.^24^ Physical activity was assessed by a structured questionnaire and expressed as daily energy expenditure in MET-h.^24, 35^ Body weight and height were measured while the subjects wore no shoes and light underwear and BMI (kg m^−2^) was calculated. Categories of BMI '<25 kg m^−2^', '25–29.9 kg m^−2^' and '⩾30 kg m^−2^' were considered as 'under/normal weight', 'overweight' and 'obese', respectively, according to WHO guidelines.^36^ Moreover, waist circumference, in cm, was measured in the middle between the twelfth rib and the iliac crest and hip circumference, in cm, was measured around the buttocks. The waist-to-hip ratio was then calculated.

#### INHES population

Participants reported the type of their profession as 'manual', 'non manual', 'housewife' (only for women), 'retired' and 'student or unemployed' and their marital status as 'single', 'married', 'separated' and 'widow'. They were also asked about their physical activity and grouped to the categories of 'physically active' or 'inactive'. Body weight and height were self-reported and BMI (kg m^−2^) was calculated. Self-reported BMI data tend to over- or under-estimate in proportion to measured BMI. Categories of BMI were calculated as above. Both physical activity and anthropometric assessment^37, 38^ were under self-reporting biases.

### Statistical analysis

#### Descriptive analysis

The normality of continuous variables was tested graphically. Continuous data are presented as mean (standard deviation) and categorical variables as frequencies. Comparisons of continuous variables between two groups of study were performed using the Student's *t*-test. Comparisons of continuous variables within more than two groups were carried out using the analysis of variance F-test. Associations between food group intake and score of adherence to MeD were tested using the Spearman's rho, while associations of categorical variables were tested using Pearson's *X*^2^-test. Two-sided *P*-value<0.05 was considered as statistically significant. STATA version 9 software was used for all calculations (STATA Corp., College Station, TX, USA).

#### Statistical modeling step 1

Crude linear regression models stratified by gender were generated with main outcome the BMI (kg m^−2^) and independent factor, the pasta consumption (grams per day) in both Moli-sani and INHES populations.

#### Statistical modeling step 2

The energy residuals methodology, previously used in the study of the association of food group intake with BMI,^39^ was used to overcome bias related to the over- or under-estimation of dietary data in both Moli-sani and INHES datasets.

In particular, linear regression was used to 'predict' individual pasta intakes on the basis of total energy intake (kcal per day), and the residual value for each regression was calculated by subtracting the observed value from the predicted value (resulting in the 'Pasta-energy residuals').

The latter was used as an independent variable in regression models with BMI (dependent variable) in crude and multi-adjusted level in both Moli-sani and INHES datasets. Multi-adjusted models were calculated by adjusting crude models for age, social status, physical activity level, energy intake and adherence to the MeD in the Moli-sani dataset; and for age, marital status, occupation, physical activity, energy intake and adherence to the MeD in the INHES dataset.

#### Statistical modeling step 3

Crude linear regression models stratified by the quintiles of body weight of the subjects were generated with BMI (kg m^−2^) as main outcome and pasta consumption (grams per day) as independent factor, in both Moli-sani and INHES populations.

As this stratification significantly affected the association of BMI with pasta intake (from positive un-stratified association to negative associations in different body weight groups after stratification), further regression analysis was performed.

Standardized residuals (that is, 'pasta-body weight residuals') were predicted in both datasets, using linear regression analysis with main outcome as pasta intake (grams per day) and independent variable as body weight (kg). The generated pasta-body weight residuals were standardized by dividing them by their estimated standard error and produced to be mathematically independent from body weight. This way of correcting pasta intake by the body weight takes into account in a generic way the individual's need.

Crude and multi-adjusted models stratified by gender were generated with main outcome the BMI (Kg m^-2^) and independent factor, the predicted pasta-body weight residuals in both Moli-sani and INHES datasets.

Multi-adjusted models were calculated by adjusting crude models for age, social status, physical activity level, energy intake and adherence to the MeD in the Moli-sani dataset; and for age, marital status, occupation, physical activity, energy intake and adherence to the MeD in the INHES dataset. Stratified linear regression analysis by groups of MeD adherence (that is, quartiles of MeD index) was performed to assess any interaction of MeD on the association of pasta with BMI.

#### Statistical modeling step 4

Linear regression analysis using the same adjustment scheme was performed with main outcome the waist or hip circumference (cm) or waist-to-hip ratio and independent factor the pasta-energy and -body weight residuals in the Moli-sani population.

#### Linear regression assumptions testing

For all models, normality of residuals, homoscedasticity and multiple co-linearity were evaluated by plotting standardized residuals against the predicted values and these assumptions tend to be fulfilled.

## Results

### Pasta intake in association with BMI

#### Moli-sani population

The prevalence of overweight and obesity was 35.7% and 27.7% in women and 50.4% and 29.0% in men, respectively (*P* for gender difference<0.001). Table 1 presents the characteristics of Moli-sani participants according to BMI group in a stratified analysis by gender. Both in women and men, the obese population was older and at lower socioeconomic status (*P* for all<0.001), had higher waist and hip circumferences and waist-to-hip ratio, and consumed more pasta (grams per day) than normal or overweight participants (*P* for both<0.05).

Pasta consumption was also associated with better adhesion to the Mediterranean diet in both genders (for both Spearman's rho>0, *P*<0.001). Among food groups included in MeD, cooked tomatoes and other sauces were strongly correlated with pasta consumption in both women (Spearman's rho=0.68 and 0.64, *P*<0.001) and men (Spearman's rho=0.71 and 0.59, *P*<0.001). Other food groups included in MeD with strong correlations with pasta intake in both genders were onions and garlic, olive oil, seasoned cheese and rice (*P* for all<0.05).

**Figure 1:**
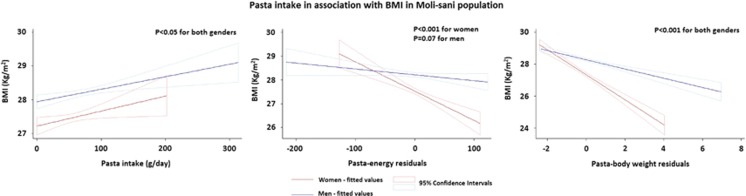
Linear regression analysis evaluating the association of pasta intake as grams per day or pasta-energy residuals or pasta-body weight residuals and BMI.

Simple linear regression analysis indicated that pasta intake expressed as grams per day was positively associated with BMI in both genders (Figure 1). However, because underreporting of energy intake was evident in obese women participants (Table 1), the energy residuals methodology was elaborated to overcome related bias and the association changed direction from positive to negative (Figure 1).

Pasta-energy residuals were negatively associated with BMI in women and not in men (Figure 1, Table 2). After adjustment for age, social status, physical activity level, energy intake and adherence to the MeD, results remained significant for women population (β-coef=−0.007, *P*=0.003 for women and β-coef=−0.001, *P*=0.58 for men).

Moreover, stratified analysis by quintiles of body weight of the subjects showed that the initial positive association of pasta intake (grams per day) with BMI changed to negative associations in the majority of body weight groups in both genders (β-coef<0, *P*<0.05), (Table 3).

Figure 1 also illustrates the change in the direction of the association of pasta intake with BMI (that is, from positive to negative) that was observed after elaborating the residuals methodology by correcting pasta intake (grams per day) by the body weight (kg) of the subjects.

Multi-adjusted linear regression analysis (Table 2) indicated that the pasta-body weight residuals were negatively associated with BMI in women and men, respectively (β-coef=−0.87, *P*<0.001 in women and β-coef=−0.51, *P*<0.001 in men) (Table 2).

Stratified analysis by MeD adherence of the association of pasta-body weight residuals with BMI showed a significant negative association in different groups in both genders (for all β-coef<0, *P*<0.05).

#### INHES population

The prevalence of overweight and obesity was 30.5% and 12.6% in women and 46.7% and 14.5% in men, respectively (*P* for gender difference<0.001). Table 4 presents the characteristics of INHES participants according to BMI group in a stratified analysis by gender. Similarly to the Moli-sani dataset, both female and male obese were older (*P* for both<0.001), at lower socioeconomic status and reported a higher intake (grams per day) of pasta in women (*P*=0.002).

Pasta intake expressed as grams per day was positively associated with BMI in women population (β-coef=0.004, *P*=0.01) and not in men (β-coef=−0.004, *P*=0.05).

Pasta-energy residuals were negatively associated with BMI in women (β-coef=−0.004, *P*=0.01) and positively in men (β-coef=0.005, *P*=0.01), in crude analysis (Table 2). These associations became non-significant after adjustments in both genders (*P*>0.05).

However, stratified analysis by the quintiles of body weight of the subjects for the association of pasta intake (grams per day) with BMI changed the initial positive association to negative associations in part of the body weight groups in both genders (β-coef<0, *P*<0.05), (Table 3).

Finally, correcting pasta intake by the body weight of the subjects, linear regression analysis showed a negative significant association of BMI with pasta-body weight residuals in both genders and in multi-adjusted models (β-coef=−0.18, *P*=0.01 in women and β-coef=−0.30, *P*<0.001 in men), (Table 2).

Stratified analysis by the group of MeD adherence of the association of pasta-body weight residuals with BMI indicated significant negative associations in different groups in both genders (β-coef<0, *P*<0.05).

### Pasta intake in association with waist and hip circumference and waist-to-hip ratio

#### Moli-sani population

Crude models indicated that pasta-energy residuals were significantly and negatively associated with waist and hip circumference in women (β-coef<0, *P*<0.05) and not in men (*P*>0.05) of the Moli-sani population (Table 5). After adjustments, only the negative association of pasta-energy residuals with hip ratio remained significant (β-coef=−0.01, *P*=0.03) (Table 5). Waist-to-hip ratio was not significantly associated with pasta-energy residuals in both genders and crude and multi-adjusted models (*P*>0.05).

Pasta-body weight residuals were significantly and negatively associated with waist and hip circumference and waist-to-hip ratio in both genders and in crude and multi-adjusted models (for all β-coef<0, *P*<0.05, Table 5).

## Discussion

Despite the evidence supporting the role of MeD adherence in the management of a favorable body weight,^21, 22, 23^ according to the best of our knowledge, there is no study evaluating this association for pasta as a MeD component. Our findings show a negative association of pasta consumption with general and central obesity in two methodologically and geographically different, large Mediterranean populations.

Pasta as a product of cereals has been since ancient times consumed in the Mediterranean area and it has been considered as one of MeD's traditional components, placed at the basis of the pyramid.^1, 2^ Our comparative analysis of data from two different Mediterranean populations supports that pasta intake is negatively associated with both indexes of obesity status and prevalence of overweight and obesity.

Our results are in agreement with a relatively recent study examining food and nutrient intakes in association with BMI in 1794 US middle-aged adults, showing that pasta intake among other food groups is negatively associated with BMI.^39^ Moreover, evidence from Greek islands supports a favorable role of carbohydrate intake on central and general obesity.^40^

On the other hand, bias of over-estimation or under-estimation presented generally in dietary data was evident also in the present datasets especially in women. As a result, pasta intake expressed as grams per day, seemed to be positively associated with BMI in crude analysis. However, to overcome the related bias, the correction of food group consumption by total caloric intake using the 'energy residual methodology' was elaborated as a relatively frequent procedure in dietary analysis.^41^ The association changed direction and from positive became negative.

To strengthen our results, we also performed multiple approaches in dietary analysis. In fact, we used both 'energy residual methodology' and residual methodology for correcting pasta intake for body weight of the subjects.

This approach was derived from the observation that in both Moli-sani and INHES datasets, the association of pasta intake as grams per day with BMI from positive became negative after stratification for the body weight of the subjects. Standardized residuals were predicted in both datasets, using linear regression analysis with main outcome pasta intake (grams per day) and independent variable body weight (kg).

The generated pasta-body weight residuals were produced to be mathematically independent from body weight. This was evident because the standardized residuals (that is, 'pasta-body weight residuals') that were predicted by using linear regression analysis with main outcome pasta intake (grams per day) and independent variable body weight (kg) are mathematically independent from body weight.

Again, pasta-body weight residuals were negatively associated with BMI in both populations and in both genders. Altogether, these different approaches strengthen the negative association of pasta intake with obesity indexes.

The measurement of body weight, as carried out in the Moli-sani study, is a simple physical examination that has limited systematic or random errors and might result in a reduction in overall error because the reported energy intake can be under miss- or over-reporting bias. The evidence that with both methodologies the negative association of pasta intake with obesity status was confirmed is possibly an indicator of the validity of the proposed methodology, although further investigation in prospective studies is needed.

During the last decades, a progressive increase in red meat consumption, fats, dairy products and simple sugars has been recorded in Italy.^10, 42^ The adherence to the MeD that may offer weight management advantages is significantly lowered.^42^ Pasta consumption has been modified, because it is frequently considered as a dietary factor that should be restricted in a weight loss program.

In this work, pasta intake was positively associated with the intake of other important food groups included in MeD such as tomatoes, tomato sauce, onions, garlic, olive oil, seasoned cheese and rice.^5, 6, 21, 22, 23^ However, it was interesting that the negative association of pasta intake with BMI was independent from MeD adherence and total daily caloric intake. Indeed, the negative association was not affected by either the addition of both MeD adherence index and total daily caloric intake into multivariate analysis or by the stratification for MeD adherence level.

From an epidemiological and clinical perspective, it could be important to evaluate the role of other food groups associated with pasta consumption on the management of body weight. The study of dietary patterns such as MeD or others not '*a-priori*' defined in association with human anthropometry would be useful to understand the effect of the combination of pasta with other food group intake on the management of body weight.

The debate of hypo-caloric high protein diets versus low fat and standard carbohydrate diets in the regulation of body weight is still on the surface of nutrition research,^15, 22, 23^ especially because health implications of high protein diets have been discussed (that is, kidney function, bone health).^17, 18, 19, 20^ Our results support that MeD, and pasta as one of its major components, should be further investigated for their role in decreasing the risk for overweight and obesity.

Another novelty of the present work is the comparative assessment of the study hypothesis in both Moli-sani and INHES datasets. In fact, the results derived from the two analyses were almost identical, despite the large differences in participant's recruitment methodology and in dietary assessment. This demonstrates the reproducibility of the present associations among studies with relative diversity in their methodologies.

In particular, the Moli-sani population is a cohort study based on face-to-face interviews and clinical examinations, while INHES is a telephone-based survey. In addition, Moli-sani recruited a representative sample of the Molise region in Italy while the population of INHES is spread all over the country. Also, the dietary evaluation was performed by using the EPIC-food frequency questionnaire in the case of Moli-sani and one 24-h dietary recall in INHES. Even the prevalence of obesity was significantly lower in INHES population because the anthropometric assessment and the age range differed. Moli-sani monitors made the measurements during the clinical assessment, while in INHES, the body weight and height were self-reported and under related bias of underreporting.

Beyond the relevance of the findings of the present work, limitations do exist. First, because both Moli-sani and INHES are cross-sectional studies, they do not allow to provide cause–effect considerations in the observed associations. Prospective anthropometric data on these populations are still missing and limit the clinical significance of the present conclusions. In addition, bias of over- or under-estimation by the use of the dietary methodologies and for the extracted data (that is, pasta intake, adherence to MeD) should be acknowledged for both studies. The co-linearity presented in food group intake data (that is, pasta intake with other food groups) limits also the present analysis.

In the Moli-sani study, although adequate from a broad epidemiological perspective, a food frequency questionnaire is less accurate at the individual level than other measurement methods. In addition, dietary information was retrieved only once and, thus, may be prone to recall bias and seasonal variation. Possible errors because of misreporting by the participating subjects should also be acknowledged. However, to rule out the possibility that the associations found were dependent on either changes in lifestyle (particularly in dietary habits) as a consequence of a disease or to the presence of less healthy food intake in healthy people, we had preliminarily excluded from our analyses all subjects with previous CVD or cancer and participants under special diets. Furthermore, the cross-sectional analysis design does not allow for concluding on the causality of the present associations.

Limitations are evident also for INHES study. The one 24-h dietary recall could not be considered as representative of the dietary habits of an individual. The telephone-based interviews are under the limitation of misreporting or under/over-estimation of food consumption. The body weight and height was self-reported and under the related bias.

In conclusion, this study for the first time has observed a negative association of pasta consumption and central and overall obesity in a large sample of Mediterranean population. Pasta as the traditional component of MeD was studied for its association with obesity indexes. The comparative assessment of two different epidemiological datasets and the similarities revealed in the studied associations provide reliability and support to the present results.

## Figures and Tables

**Table 1:** Distribution of various characteristics of Moli-sani participants according to BMI group

N=*14 402*	*Women (*N=*7216)*				*Men (*N=*7186)*			
	*Under/normal weight*^a^ (N=*2643)*	*Overweight (*N=*2575)*	*Obese (*N=*1998)*	P*-value*^b^	*Under/normal weight*^a^ (N=*1479)*	*Overweight (*N=*3621)*	*Obese (*N=*2086)*	P*-value*^b^
Age (years)	49 (10)	54 (11)	57 (11)	<0.001	52 (12)	53 (11)	55 (11)	<0.001
Socioeconomic status (score 0–8)	3.89 (1.38)	3.40 (1.34)	3.11 (1.31)	<0.001	3.67 (1.40)	3.53 (1.36)	3.37 (1.32)	<0.001
Physical activity level (Mets-hour)	42.5 (7.1)	43.0 (8.1)	43.3 (8.9)	0.002	44.3 (9.8)	44.5 (10.5)	44.3 (10.6)	0.65
Waist circumference (cm)	80.5 (7.7)	91.5 (7.9)	105 (10.3)	<0.001	86.7 (5.9)	95.5 (5.7)	108 (8.4)	<0.001
Hip circumference (cm)	95.4 (4.9)	103 (5.2)	114 (9.2)	<0.001	96.0 (4.6)	102 (4.8)	110 (7.2)	<0.001
Waist-to-hip ratio	0.84 (0.07)	0.89 (0.07)	0.92 (0.08)	<0.001	0.90 (0.05)	0.94 (0.05)	0.98 (0.05)	<0.001
Energy intake (kcal per day)	2070 (551)	1998 (533)	1964 (542)	<0.001	2417 (664)	2398 (645)	2451 (707)	0.02
Adherence to MeD (Italian score 0–11)	3.97 (1.79)	3.95 (1.74)	3.94 (1.72)	0.85	3.72 (1.76)	3.80 (1.74)	3.95 (1.74)	<0.001
Pasta (grams per day)	57.3 (30.4)	57.1 (28.9)	59.2 (30.8)	0.03	75.6 (39.1)	75.1 (37.9)	78.5 (39.5)	0.004
Pasta (g kcal^−1^ of daily energy intake)	0.028	0.029	0.031	<0.001	0.0318	0.0317	0.0325	0.13

Abbreviation: BMI, body mass index.

a The number of underweight individuals was very low and did not affect the means of normal weight individuals (i.e., <1% of total population).

b *P*-value derived through comparisons of continuous characteristics between BMI groups using one-way analysis of variance F-test and results are presented as mean (standard deviation).

**Table 2:** Linear regression analysis evaluating the association of pasta consumption with BMI in Moli-sani and INHES participants^a^

	*Moli-sani population (*N=*14 402)*	
	*Women (*N=*7216)*	*Men (*N=*7186)*
*Pasta-energy residuals*		
Unadjusted models	−0.012 (<0.001)	−0.002 (0.07)
Multi-adjusted models^b^	−0.007 (0.003)	−0.001 (0.58)
*Pasta-body weight residuals*^c^		
Unadjusted models	−0.78 (<0.001)	−0.29 (<0.001)
Multi-adjusted models^b^	−0.87 (<0.001)	−0.51 (<0.001)
	*INHES population (*N=*8964)*	
	*Women (*N=*4782)*	*Men (*N=*4182)*
*Pasta-energy residuals*		
Unadjusted models	−0.004 (0.01)	0.005 (0.01)
Multi-adjusted models^d^	−0.001 (0.36)	0.002 (0.05)
*Pasta-body weight residuals*^e^		
Unadjusted models	−0.08 (0.25)	−0.40 (<0.001)
Multi-adjusted models^d^	−0.18 (0.01)	−0.30 (<0.001)

Abbreviation: BMI, body mass index.

a Results derived from linear regression analysis with main outcome the BMI (kg m^−2^) and independent variable the pasta-energy residuals or pasta-body weight residuals. Results are presented as β-coefficients (*P*-value) (for 1 unit increase in predicted residuals).

b Models have been adjusted for age, socioeconomic status, physical activity level, energy intake and Mediterranean pattern adherence.

c The β-coefficient for 1 unit increase in pasta-body weight residuals corresponded to 35 g per day increase in pasta intake.

d Models have been adjusted for age, profession type, marital status, physical activity, energy intake and Mediterranean pattern adherence.

e The β-coefficient for 1 unit increase in pasta-body weight residuals corresponded to 48 g per day increase in pasta intake.

**Table 3:** Linear regression analysis stratified by body weight evaluating the association of pasta consumption (grams per day) with BMI in Moli-sani and INHES participants^a^

	*Moli-sani population (*N=*14* *402)*				
	*Q1 (<62* *kg)*	*Q2 (62–70* *kg)*	*Q3 (70–77* *kg)*	*Q4 (77–86* *kg)*	*Q5 (>86* *kg)*
*Unadjusted models*					
* *β-coef for 35 g per day increase in pasta intake	−0.11 (0.02)	−0.34 (<0.001)	−0.37 (0.001)	−0.44 (<0.001)	−0.37 (<0.001)
					
*Multi-adjusted models*^b^					
* *β-coef for 35 g per day increase in pasta intake	−0.01 (0.84)	−0.17 (0.001)	−0.19 (0.002)	−0.25 (<0.001)	−0.23 (0.01)
	*INHES population (*N=*8964)*				
	*Q1 (<60* *kg)*	*Q2 (60–67* *kg)*	*Q3 (67–75* *kg)*	*Q4 (75–83* *kg)*	*Q5 (>83* *kg)*
*Unadjusted models*					
β-coef 48 g per day increase in pasta intake	0.07 (0.16)	−0.001 (0.99)	−0.18 (0.001)	−0.01 (0.82)	−0.43 (0.03)
					
*Multi-adjusted models*^c^					
β-coef for 48 g per day increase in pasta intak*e*	0.06 (0.19)	0.03 (0.57)	−0.18 (<0.001)	0.02 (0.76)	−0.20 (0.04)

Abbreviations: BMI, body mass index.

a Results derived from linear regression analysis with main outcome the BMI (kg m^−2^) and independent variable pasta intake (grams per day) and are presented as β-coefficients (*P*-value).

b Models have been adjusted for age, socio-economic status, physical activity level, energy intake and Mediterranean pattern adherence.

c Models have been adjusted for age, profession type, marital status and physical activity, energy intake and Mediterranean pattern adherence.

**Table 4:** Distribution of various characteristics of women and men INHES participants according to BMI group^a^

N=*8964*	*Women (*N=*4782)*				*Men (*N=*4182)*			
	*Under/normal weight (*N=*2723)*^b^	*Overweight (*N=*1458)*	*Obese (*N=*601)*	P*-value*^c^	*Under/normal weight (*N=*1622)*^b^	*Overweight (*N=*1952)*	*Obese (*N=*608)*	P*-value*^c^
Age (years)	53 (16)	60 (13)	61 (12)	<0.001	53 (17)	59 (14)	58 (13)	<0.001
*Profession type (%)*				<0.001				<0.001
Manual	13.0	12.4	15.3		22.8	21.7	25.4	
Non manual	36.5	19.6	15.1		36.1	30.8	27.5	
Housewife	17.4	23.7	25.5		Omitted	Omitted	Omitted	
Retired	25.4	40.9	41.3		31.6	43.9	43.2	
Student/ Unemployed	7.7	3.4	2.7		9.5	3.5	4.0	
*Marital status (%)*				<0.001				<0.001
Single	20.8	9.2	6.9		24.3	11.1	10.0	
Married	67.6	76.4	74.7		72.5	84.9	83.9	
Separated	2.7	1.2	2.0		1.6	1.7	1.8	
Widow	8.9	13.2	16.4		1.6	2.4	4.3	
Physically active (%)	22.0	12.8	8.7	<0.001	29.2	17.1	8.4	<0.001
Adherence to MeD (Italian score 0–11)	3.61 (1.77)	3.71 (1.73)	3.82 (1.70)	0.02	3.89 (1.74)	3.98 (1.66)	4.01 (1.74)	0.17
Energy intake (kcal per day)	1789 (632)	1777 (622)	1842 (763)	0.10	2089 (712)	2059 (742)	2157 (765)	0.02
Pasta (grams per day)	49.4 (46.1)	52.1 (46.1)	56.4 (50.8)	0.002	64.7 (48.9)	62.7 (49.1)	64.6 (48.7)	0.44
Pasta (g kcal^−1^ of daily energy intake)	0.029	0.031	0.032	0.02	0.033	0.033	0.032	0.52

Abbreviation: BMI, body mass index.

a Results are presented as mean (standard deviation) for continuous variables and as frequencies for categorical data.

b The number of underweight individuals was very low and did not affect the means of normal weight individuals (i.e., <1% of total population).

c *P*-value derived through comparisons of continuous and categorical variables between BMI groups using the one-way analysis of variance F-test and Pearson's *X*^2^-test, respectively.

**Table 5:** Linear regression analysis evaluating the association of pasta consumption with waist and hip circumference and waist-to-hip ratio in Moli-sani participants^a^

	*Women (*N=*7216)*	*Men (*N=*7186)*
*Waist circumference (cm)*		
Pasta-energy residuals		
Unadjusted models	−0.02 (<0.001)	−0.01(0.05)
Multi-adjusted models^b^	−0.009 (0.12)	−0.003 (0.48)
Pasta-body weight residuals^c^		
Unadjusted models	−1.8 (<0.001)	−0.7 (<0.001)
Multi-adjusted models^b^	−2.0 (<0.001)	−1.2 (<0.001)
		
*Hip circumference (cm)*		
Pasta-energy residuals		
Unadjusted models	−0.02 (<0.001)	−0.003 (0.18)
Multi-adjusted models^b^	−0.01 (0.03)	−0.0001 (0.97)
Pasta-body weight residuals^c^		
Unadjusted models	−1.5 (<0.001)	−0.6 (<0.001)
Multi-adjusted models^b^	−1.7 (<0.001)	−1.0 (<0.001)
		
*Waist-to-hip ratio*		
Pasta-energy residuals		
Unadjusted models	−0.0001 (0.06)	−0.0001 (0.09)
Multi-adjusted models^b^	−0.00001 (0.95)	−0.00002 (0.24)
Pasta-body weight residuals^c^		
Unadjusted models	−0.005 (<0.001)	−0.002 (0.004)
Multi-adjusted models^b^	−0.005 (<0.001)	−0.003 (<0.001)

a Results derived from linear regression analysis with main outcome as the waist or hip circumference (cm) or waist-to-hip ratio and independent variable as the pasta-energy residuals or pasta-body weight residuals. Results are presented as β-coefficients (*P*-value) (for 1 unit increase in predicted residuals).

b Models have been adjusted for age, socioeconomic status, physical activity level, energy intake and Mediterranean pattern adherence.

c The β-coefficient for 1 unit increase in pasta-body weight residuals corresponded to 35 g per day increase in pasta intake.
